# Mature Adults at the GP: Length of Visit and Patient Satisfaction—Associations with Patient, Doctor, and Facility Characteristics

**DOI:** 10.3390/medicina58020159

**Published:** 2022-01-20

**Authors:** Marta Rzadkiewicz, Gorill Haugan, Dorota Włodarczyk

**Affiliations:** 1 Department of Health Psychology, Medical University of Warsaw, Litewska 14/16, 00-575 Warsaw, Poland; dwlodarczyk@wum.edu.pl; 2 Department of Public Health and Nursing, Norwegian University of Science and Technology, P.O. Box 8905, 7491 Trondheim, Norway; gorill.haugan@ntnu.no; 3 Faculty of Nursing and Health Science, Nord University, 7600 Levanger, Norway

**Keywords:** length of the visit, patient satisfaction, health status, appointments and schedules, general practice, adult, aging, primary care, physicians

## Abstract

*Background and objectives*: The consultation time for more mature adults is often perceived as longer, increasing with the patient’s age and boosting their satisfaction with the visit. However, factors determining patient satisfaction (PS) or the consultation time (CT) in the population aged 50+ are not clearly identified. A cross-sectional design was used to identify factors specific to the facility (e.g., size, staff turnover), doctor (e.g., seniority, workload), and patient (e.g., self-rated health, impairment of activities) that are related to PS and the CT. Our secondary focus was on the relation of PS to the CT along with the role of the patient’s age and gender for both. *Materials and Methods*: Doctors (*n* = 178) and their 1708 patients (aged 50–97) from 77 primary care facilities participated in the study. The Patient Satisfaction with Visit Scale score and the CT were the outcome measures. *Results*: We identified associations with the CT in terms of the facility-related factors (number of GPs, time scheduling); doctors’ workload and health; and patients’ education, time attending GP, and impairments. PS was additionally governed by doctors’ perceived rate of patients aged 65+, as well as the patients’ hospitalization in the prior year, frequency of visits, and impairments. For adults aged 50+ the CT was unrelated to PS and both remained independent of patients’ age. *Conclusions*: Specific factors in terms of the facility, GP, and patient were identified as related to PS and the CT for participating adults in primary care. During visits of patients aged 50+ at their GP, there is scope for both time-savings and patient satisfaction improvements, when paying attention, e.g., to the time scheduled per visit, the number of doctors employed, and the patients’ impairments.

## 1. Introduction

Patient satisfaction (PS) among adults in primary care increases with age and, on average, reflects positive experiences [1,2]. Factors determining PS are identified as representing three main domains related to the facility [3,4,5], doctor (including the patient-doctor relationship) [5,6,7], and patient [4,8]. Established correlates of satisfaction are represented, for example, by lower staff turnover [3,4] or facility size [9]; the GP’s gender, workload, and work satisfaction [2,6]; or the patient’s age, health status, or the continuity of the GP’s care [1,8].

Many of the PS determinants are potential predictors of the consultation time (CT). Thus, exploration of this context could provide important organizational information for primary care. In turn, the CT indicates the quality of care associated with health outcomes; hence its association with PS is worth considering [6,10,11]. However, here too, characteristics of a patient (e.g., multimorbidity, deprivation status [12,13]), doctor (e.g., gender, workload, and experience [11,12]), or facility often explain the CT better than the nature of the medical problem a patient presents with [8,14]. 

At the same time, reports remain ambiguous regarding the relationship between the CT at the GP and patients’ age [12,13,15], or between PS and their self-rated health (SRH) [13,14], yet only a handful of studies cover a wider range of CT and/or PS predictors [10,12]. Studies examining associations between the CT and PS also report discrepant findings—from none to strong [6,10,11,15,16,17,18]. Moreover, we thought that studies illustrating the above relationships are particularly important, yet they are scarce when it comes to more mature primary care users.

Primary care in Poland—with its healthcare system based on health insurance and a free choice of physician (which is similar to the British and Scandinavian systems)—is facing the same demographic trends as across Europe. The population of seniors is growing rapidly, but a gap still exists in identifying the CT and/or PS determinants among mature adults. Understanding these associations is important for the efficiency and quality of primary care.

### Objective

To investigate what determines PS and the CT in relation to GP consultation, we considered factors from three domains characterizing facilities, doctors, and patients. To the best of our knowledge, analyses encompassing such a broad perspective on PS and CT determinants in primary care are scarce, and such outcomes have not been assessed among adults aged 50+ in contemporary European society. Thus, we analyzed them both contextually (separately for each domain of factors) and comprehensively (including mutual relationships between factors from different domains). The following three research questions were analyzed in parallel in this cross-sectional study:

1. With regard to the facility, the physician, and the patient, which characteristics determine PS with a visit to the GP and the CT among mature adults?
2. How does PS with a visit to the GP relate to the CT in this study group?
3. Does a patient’s age or gender diversify PS and/or the CT?

The main aim of the research was to identify factors jointly affecting PS and the CT as a potential focus of efforts to improve the quality and cost-effectiveness of primary care practice for aging adults.

## 2. Materials and Methods

**Figure 1:**
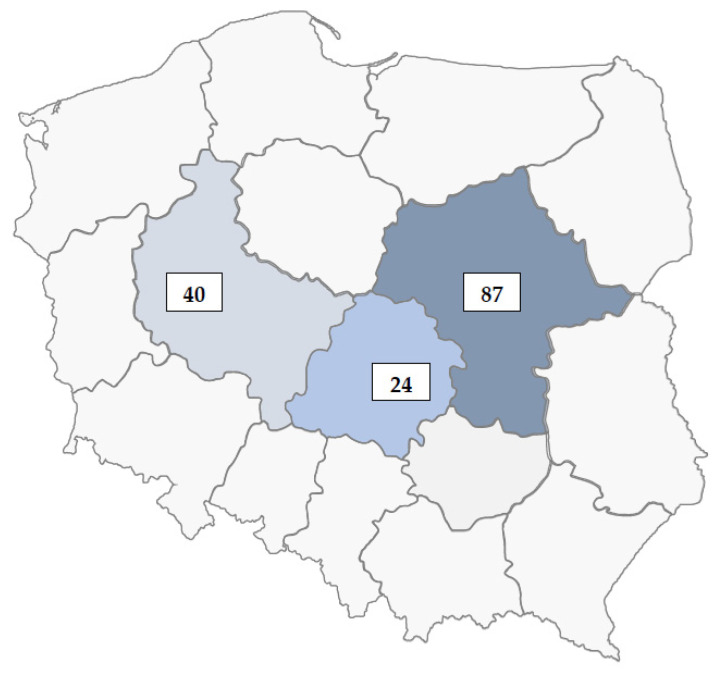
Map of Poland with marked districts where PHFs were involved in PRACTA study. Out of all 151 PHFs participating, 77 were randomly chosen for present analysis.

The present data came from the PRACTA—activating the elderly in medical practice—research project, conducted between 2013 and 2016 in primary healthcare facilities (PHFs) located in central Poland (Figure 1), [19,20]. Using a cross-sectional design, PS and the CT were analyzed with reference to selected characteristics of the PHFs (*n* = 77), GPs (*n* = 178), and their patients aged 50+ (*n* = 1708; with response rates of 20%, 50%, and 74.6%, respectively). The PHF-related information and agreement were obtained in advance. The patient inclusion criteria were: aged 50+, had an appointment with a participating GP on the given day, and had the ability to fill in the questionnaire. The GPs were approached earlier on in their offices; and patients were approached in the PHF waiting room before and directly after the appointment. The institutional bioethics committee approved this study; all participants signed the informed consent form to participate. 

### 2.1. Independent Variables

PHF-specific determinants were rated by the managing staff and included: the organizational structure (state/privately owned); location; staff turnover (1 = *very high* to 5 = *very low*); time formally scheduled per visit; number of GPs employed; and average number of patients seen daily at the clinic. 

All doctor-specific and patient-specific independent variables are presented in detail in Table 1. Since we aimed to capture the effect of aging on the CT and PS, the Doctors’ Work Satisfaction scale (Appendix A) concerned their experience with the care of adults aged 65+. The starting phrase was: “*Regarding only your work with elderly patients (65+), please respond to the following statements*…”. This scale contains five items and was inspired by the Satisfaction with Life Scale [21], but modified to reflect professional experiences (e.g., *The conditions of my work are excellent*) [22]. The Cronbach’s alpha reliability coefficient (*α* = 0.78) was good in this study. We expected that doctors gaining more satisfaction when working with seniors would have more satisfied patients post visit, and we were keen to explore the relationship between work satisfaction and the CT. 

For patients, the Health Impact on Activities (HIA) scale was used [23], (Appendix B), describing ten everyday activities evaluated on a four-point scale, indicating the level of restraint and showing good reliability (*α* = 0.95).

### 2.2. Outcome Variables

To assess PS, the PRACTA Patients’ Satisfaction with Visit Scale was developed, consisting of seven items (e.g., “Would you recommend this doctor to your family/friends?”) scored on a seven-point scale (Appendix C). A higher score indicated a higher PS (reliability coefficient *α* = 0.93). The CT was noted in minutes immediately post visit.

### 2.3. Statistical Analysis

A generalized linear model—GENLIN, distribution Gamma, link identity [24]—was applied using IBM SPSS 24 software to test the determinants of PS and CT, separately for each outcome. First, groups of factors specific to PHFs, GPs, and patients were analyzed contextually. Next, all factors identified as significant in contextual models entered one comprehensive model, separately for the CT and PS. Effects with a significance value of 0.05 or lower were presented.

## 3. Results

### 3.1. Descriptive Statistics

Some 59.2% of the participating PHFs were public, mostly located in big towns (M = 4.41, SD = 1.64, range 1 to 6), employed more than five GPs (M = 5.74, SD = 3.37, range 1–20), and admitted M = 156.27 (SD = 84.65, range 25–400) patients per day. The staff turnover was rated between *average* and *low* (M = 3.85, SD = 0.76), and the time assigned per visit was M = 13.11 min (SD = 2.80, range 7–20). Table 1 and Table 2, respectively, present the descriptive statistics for GPs and their patients.

On average, the GPs were in their fifties, had more than 20 years of professional experience, and worked more than 40 hours a week. Nevertheless, doctors’ SRH was often good, as was their satisfaction with their work with patients aged 65+. Despite the fact that doctors declared that the proportion of their patients aged 65+ usually exceeded 50%, only some of the GPs had geriatric training.

Patients’ mean age approached 70 years, two-thirds were married, and nearly one fifth had a higher level of education or were living alone. The majority had been hospitalized during the previous year and had presented to a GP for a medical reason.

PS showed a mean of M = 5.68 with SD = 0.82 and a range of 2.29–7.00. The average CT was 15.83 min (SD = 5.81, range 1–90), which is comparable with other countries [3]. To answer the first research question on the CT and PS, three groups of specific determinants of the CT and PS were screened independently.

### 3.2. Contextual Analysis—Determinants of CT

Facility-specific predictors yielded relationships with the CT (Wald’s *χ*^2^ = 78.727, *p* < 0.001 for the model; the intercept in the model was 177.091, *p* < 0.001) in terms of the number of GPs, staff turnover, and time scheduled per visit (relevant detailed statistics for this paragraph are available in Appendix D).

Among the doctor-related variables, the self-rated health was the most notable predictor of the CT, with healthier doctors spending less time with their patients. Additionally, a set of other factors was also associated with the CT: the doctors’ training in geriatrics, professional seniority, seniority within the facility, working hours in the facility, hours worked in total, and satisfaction with their work with seniors. That model yielded Wald’s *χ*^2^ = 69.691, *p* < 0.001, with an intercept equal to 130.078, *p* < 0.001.

Patient-related factors that were associated with the CT include (Wald’s *χ*^2^ = 75.730, *p* < 0.001 for the model; the intercept in the model was 59.189, *p* < 0.001) their education, hospital use in the last 12 m, HIA, waiting time, and attendance in years.

### 3.3. Contextual Analysis—Determinants of PS

Facility-specific predictors yielded relationships with the CT (Wald’s *χ*^2^ = 78.727, *p* < 0.001 for the model; the intercept in the model was 177.091, *p* < 0.001) in terms of the number of GPs, staff turnover, and time scheduled per visit (relevant detailed statistics for this paragraph are available in Appendix D).

Among the doctor-related variables, the self-rated health was the most notable predictor of the CT, with healthier doctors spending less time with their patients. Additionally, a set of other factors were also associated with the CT: their training in geriatrics, professional seniority, seniority within the facility, working hours in the facility, working hours in total, and satisfaction with their work with seniors. That model yielded Wald’s 69.691, *p* < 0.001; with an intercept equal to 130.078, *p* < 0.001.

Patient-related factors that were associated with the CT include (Wald’s *χ*^2^ = 75.730, *p* < 0.001 for the model; the intercept in the model was 59.189, *p* < 0.001) patients’ education, hospital use in the last 12 m, HIA, waiting time, and attendance in years.

### 3.4. Comprehensive Analysis—Determinants of CT and PS

#### 3.4.1. Consultation Time

Three determinants from each domain—facility, doctor, and patient—predicted the CT in the comprehensive model (Table 3). The turnover of staff, number of doctors, and time scheduled per visit were the most significant PHF-specific predictors of CT. The visits were shorter when the staff turnover was lower, the number of GPs higher, and the scheduled time shorter. The strongest CT predictor among the GPs’ features was their SRH—worse doctor’s health increased the CT. Additionally, doctors who were working more hours weekly in a given facility spent more time with their patients, while those working more hours in general did the opposite. The comprehensive model did not confirm the effects of doctors’ professional seniority, training in geriatrics, and satisfaction with their work with mature patients, which were observed in the contextual analysis. The level of impairment (a higher HIA score increasing the CT), education (a reversed relationship), and years of attendance with a specific GP represented the patient-specific predictors.

#### 3.4.2. Patient Satisfaction

For PS, among facilities’ characteristics, the location, number of working GPs, and the time scheduled per visit showed significant effects, with smaller populations, teams, and shorter times increasing this outcome. Among GP-specific features, the GP’s marital status predicted PS, although no particular differences were found (all *p* > 0.06). The GPs reporting more patients aged 65+ received lower PS scores. Their seniority in a given facility, work satisfaction, and SRH had a meaning in the contextual models only. Patient characteristics showed the most complex influence on PS. A non-medical aim of the visit, stronger HIA, no hospitalizations, fewer visits last year, longer waiting time, and less difficulty scheduling a visit all contributed to a lower PS.

The effect of the CT on PS was feeble—with the β value explaining less than 1% of the variance—in the contextual model, but it disappeared in the comprehensive model. This indicates no or only a marginal association between the CT and PS among mature PHF users (the second research question). A patient’s age or gender was not found to predict the CT or PS (all Wald’s *χ*^2^ < 1.09, *p* > 0.25; the third research question).

According to the main aim of the study, we found that the time formally scheduled per visit, the number of GPs in the PHF, and the health impact on a patient’s activities jointly determined both outcomes (the CT and PS) within the comprehensive analyses. To summarize, in terms of the PHF’s features, the comprehensive analyses of both PS and the CT confirmed the contextual effects. The GP-specific characteristics showed different effects on the CT and PS. Among patient characteristics, more health-related variables and perceived organizational aspects of the visit predicted PS than the CT in the comprehensive model.

## 4. Discussion

Care-related organizational aspects (the PHF location, number of GPs, time scheduled per visit, patient-rated waiting time, and easiness of scheduling an appointment) and patients’ health-related variables (their HIA, hospital use, and frequency of attending GP in the last 12 months) appeared to be the two most prominent groups of variables determining PS with visits. Thus, this study contributes to the conceptualization of PS, which is still considered incomplete [25,26]. Determinants of the CT appear to reflect the continuity of care (years attending a given GP, staff turnover), PHF status (the number of GPs, time scheduled per visit), the GP’s professional profile (seniority and workload) and health, and patient’s disabilities (HIA). Some similar effects were found in other studies [12,13,27]. Notably, the effect on both analyzed outcomes was observed for the consultation time a PHF schedules per visit, the number of GPs in a PHF, and patients’ health-related impairments (HIA).

### 4.1. Knowledge from Negative Results

It is important to point out that some effects that did not appear in the present study could shed light on PS and the CT in general practice. We found PS among aging adults to be relatively independent of the CT, representing new knowledge about this group of patients, yet complementing other research where similar results were observed [10,15,27,28]. Interestingly, in one study [18] the link between the CT and PS was found mainly for a CT of 15 min or less, which may explain the lack of effect over a broader time scope. Among factors featuring PHFs and GPs, some observations can be interpreted as meaningful due to their lack of statistical significance. The source of facilities’ funding, the GP’s professional seniority, and their training in geriatrics had no effect in the comprehensive models on either outcome. This partially confirms existing findings [27], yet warrants further investigation exploring, for example, why a GP’s greater experience and work satisfaction matter only in the contextual analysis.

No relationship was observed between patients’ age, gender, or SRH in the comprehensive analysis of the CT or PS, unlike in some earlier studies [2,4], yet confirming another [13]. This might be due to the large number of competing factors and/or the fact that our group had a significantly narrower age range, making it more homogeneous. In a study of adults aged 65+ only, the CT with a GP was comparable with samples including younger and mature patients [14], which is in line with our observation. Nevertheless, the present finding that neither the CT nor PS increased with mature patients’ age stands in contrast to some other research [12] and stereotypes, where physicians describe visits by mature adults as more time-consuming, running more slowly, and boring [29,30,31]. Another common claim, that lonely senior patients tend to seek more social than medical support, was partially contested, since neither the CT nor PS increased for those living alone or among widowers, a finding similar to one recently reported by Oser and co-workers [32]. However, we did not analyze the content of the visit [27], and the measure used here only partially indicates loneliness [33].

### 4.2. Strengths and Limitations

Based on one large sample of mature adults taken from a basic level of healthcare, this study bridges the gap between earlier studies in which the CT and PS were usually investigated separately, and the range of their determinants has rarely encompassed all three domains related to facility, doctor, and patient. For example, this study confirms the role of the continuity of care not only for PS [33], but also for the CT, and does so similarly (in the contextual model) for GPs’ work satisfaction and retention [10,11].

A twofold—contextual and comprehensive—analysis shows the meaning of specific factors in a narrower or broader context. While some of the included variables have been studied previously—this study benefits from putting them together—others were not previously analyzed in terms of PS (physician’s training, rate of elderly patients) or the CT (GP’s work satisfaction or staff turnover).

Despite the fact that we applied a comprehensive approach with multiple variables, many potential complex interactions (e.g., between the CT and treatment effectiveness) still need to be understood before more definitive steps can be taken. The present data were collected from three independent sources, but were mostly self-reported. This, along with the cross-sectional design, represents a limitation of this study in terms of dynamics and objective medical records. Owing to the aforementioned shortcomings, the present results should be generalized carefully.

### 4.3. Implications for Research and/or Practice

The World Health Organization promotes the adaptation of primary healthcare to the needs of the growing population of mature adults [34], together with actions towards active aging [35]. Nevertheless, only a very small number of research findings in the field get used in healthcare management [36]. The present results can serve as a source of evidence-based knowledge to be implemented in PHFs, but also as an inspiration for further research. Among other things, this study shows that the number of employed GPs and staff turnover, if controlled, can help to optimize the quality of primary care in terms of CT and PS. The management of PHFs focused on doctors can consider their professional seniority, satisfaction from work with adults aged 65+, and SRH, for both the CT and PS as shown in the contextual analysis. As regards patient-specific factors, the adjustment of PHFs should acknowledge the role of educational level, HIA, and perceived accessibility (waiting time, ease in scheduling, prospect of attending the same GP) for both the CT and PS. Neither the CT nor PS increased with the patient’s age, and the CT was a factor only marginally predicting PS. This finding can be important for PHFs’ organizational functioning, e.g., in scheduling appointments. Respecting doctor–patient interaction as a predictor of PS [7,37], the present study complements the knowledge that can serve the medical education well. Training in geriatrics especially might benefit from enriching GPs’ skills in competencies to meet more mature patients’ needs [20], thus enhancing PS among seniors. To summarize the practical approach, the target at which the intervention is aimed (e.g., only for doctors; or integrating effects on the facility, doctors, and patients) should be considered carefully. Depending on this choice, units responsible for the relevant programs may use the results of the contextual or comprehensive analysis of the present study.

Additionally, our study permits the comparison of a range of various measures of patient-reported health status showing that, depending on the choice, using them can allow substantially different interpretations. Interestingly, a patient’s SRH and declared number of diseases were unrelated to PS and the CT, whereas the health impact on activities was an eminent determinant of both. One possible reason for this discrepancy is that only the last scale has multiple items and relates closely to everyday life. This observation explains, to some extent, the existence of other contradictory findings in this area where the patient-reported health status shows both a positive [11] and a negative relationship with PS [4].

The suggestions for further research extend from finding effective means for improving the PHFs’ participation rates, through the careful choice of tools measuring subjective health status, to studies analyzing the CT and PS with respect to treatment effectiveness, which should consider specific variables presented here. Most notably, a valuable development of the above approach would be a set of research including objective medical records and a prospective design analyzing described predictors.

## 5. Conclusions

Participants’ health-related factors and care-related organizational aspects were found to be particularly important for PS. Patients aged 50+ with health problems resulting in limited activity experienced their visits to the GP as less satisfying, as did those who were less frequent users of medical care. Allowing the continuity of the patient–physician relationship might help to improve not only patients’ satisfaction but also GPs’ time management. The age of patients aged 50+ did not predict either PS or the CT and the consultation length was unrelated to satisfaction with it in this group, which opposes the common stereotype concerning the needs of mature patients. If considered, many of the mentioned factors, such as the size of a practice, a physician’s workload, and the degree of a patient’s impairment, permit some organizational adjustment. Therefore, they should be monitored to enhance the cost-effectiveness and quality of primary care.

Key points

∗ The timing of, and patient satisfaction with, appointments with a GP were linked to facility-, doctor-, and patient-related characteristics, with little comprehensive research.
∗ Mature patients with health problems causing impairments and those less frequently using medical care could experience lower satisfaction.
∗ Allowing the continuity of the patient–GP relationship could improve both a patient’s satisfaction and a GP’s time schedule.
∗ Satisfaction and the length of visits appeared unrelated and were both independent of patients’ age for patients aged 50+.
∗ The nature of the relationship between the patient’s subjective health status and PS or the CT might differ according to the health measurement used.

## Figures and Tables

**Table 1:** Descriptive variables related to physicians.

Characteristic of Physicians (*n* = 178)	Scale, Range, or Units	Statistics	Remarks
Age	Range 27–87	M = 50.14, SD = 1.64	
Gender		64.6% women	
Marital status	4 choices	81% married	
Geriatric training	0 = *none*, 1 = *once*, 2 = *many times*	M = 0.75, SD = 0.75	Majority had no training
Declared rate of patients aged 65+ last year	From 1 = *about 25%* to 4 = *more than 75%*	M = 2.78, SD = 0.73	The mean is close to: *more than 50%, but less than 75%* answer
Professional seniority	Range 1–60 years	M = 23.60, SD = 12.60	
Seniority in given PHF	Range 1–50	M = 12.88, SD = 12.79	
Hours of work/week	Range 8–72	M = 42.29, SD = 10.61	
Hours in PHF/week	Range 4–50	M = 33.05, SD = 10.73	
Self-rated health	Range 1–4	M = 1.83, SD = 0.70	Scale from 1 = *very good* to 5 = *very poor*
Satisfaction of work with seniors	Range 1.00 to 7.00	M = 5.11, SD = 1.06	Five items (scale from 1 = *strongly disagree* to 7 = *strongly agree*)

PHF: primary healthcare facility; M: mean; SD: standard deviation.

**Table 2:** Descriptive variables related to patients.

Characteristics of Patients (*n* = 1708)	Answer Examples, Range	Mean, SD, or % (*n*)	Remarks
Age	Range 50–97	M = 69.59, SD = 8.98	
Gender		57.3% (960) women	
Education	1 = *primary* to 5 = *higher*	M = 3.31, SD = 1.29	19.4% with higher
Marital status	4 choices	63.4% (1061) married	Marriage/partnership, single, widowed, separated/divorced
Living not/alone		19.1% (326) alone	
Work status	5 choices	7% (119) working	
Financial status	1 = *poor* to 5 = *good*	M = 3.21, SD = 0.77	M indicates above *average*
Hospital use the year prior	Yes/no	71.3% (1194) used hospital	Included emergency room visits, observation, etc.
Non/medical visit	Medical/other	84.6% (1417) came for a medical reason	*Other* means formal (e.g., documents, reference) reasons
Self-rated health	1 = *very good* to 5 = *very poor*	M = 2.88, SD = 0.72	M indicates close to *average*
Number of diseases	0 = *none* to 4 = *4 or more*	M = 1.51, SD = 0.83	M falls between *one* and *2–3 diseases*
Health Impact on Activities (HIA)	Mean score range 1–4	M = 1.45, SD = 0.60	10 items (scale from 1 = *doesn’t limit at all* to 4 = *limits very much)*
Waiting time	1 = *same day* to 5 = *more than 2 weeks*	M = 1.89, SD = 1.06	M indicates close to *up to 3 days*
Ease of scheduling	1 = *very easy* to 5 = *very difficult*	M = 2.29, SD = 0.85	M indicates less than *easy*
Length of attendance	Year range 0–40	M = 6.41, SD = 7.04	0 means *first visit*
Visits last 12 months	1 = *Not at all* to 4 = *8 or more times*	M = 1.48, SD = 0.75	50.6% indicated *2 or less visits*

**Table 3:** Significant facility-, factor-, and patient-specific determinants of PS and CT. The results from GENLIN modeling for comprehensive models (patient-specific variables after inclusion of facility-specific and doctor-specific factors).

	Patient Satisfaction		Consultation Time	
	Wald’s *χ*^2^ (*β*)	Wald’s CI ^†^	Wald’s *χ*^2^ (*β*)	Wald’s CI ^†^
Facility characteristic				
Location	21.000 *** (−0.060)	−0.086 to −0.034		
Number of GPs	8.571 *** (−0.015)	−0.024 to −0.005	5.371 * (−0.085)	−0.157 to −0.013
Staff turnover			17.585 *** (−0.892)	−1.309 to −0.475
Time reserved per visit	33.589 *** (−0.043)	−0.057 to −0.028	4.086 * (0.119)	0.004 to 0.235
Doctor characteristic				
Marital status	9.529 *			
Self-rated health			13.219 *** (0.725)	0.334 to 1.116
% patients aged 65+	16.417 *** (−0.095)	−0.141 to −0.049		
Working hours general			5.626 * (−0.032)	−0.058 to −0.006
Working hours facility			18.932 *** (0.052)	0.029 to 0.076
Patient characteristic				
Education			7.084 ** (−0.309)	−0.535 to −0.081
Visit aim: medical	15.168 *** (−0.043)			
Hospital use	7.179 ** (−0.112)	−0.193 to −0.030		
HIA	13.409 *** (−0.136)	−0.208 to −0.063	6.329 ** (0.583)	0.129 to 1.037
Waiting time for visit	121.564 *** (−0.216)	−0.255 to −0.178		
Easicly scheduled	19.587 *** (−0.289)	−0.122 to −0.456		
Attendance years			4.625 * (−0.046)	−0.088 to −0.004
Attendance 12 m	60.884 *** (0.228)	0.171 to 0.285		

^†^ All CIs refer to Wald’s 95% evaluation, *** *p* ≤ 0.005, ** *p* ≤ 0.01, and * *p* ≤ 0.05. The Wald’s *χ*^2^ values for the comprehensive models concerning the CT and PS are *χ*^2^ = 149.793 *** and *χ*^2^ = 452.333 ***, respectively; HIA—Health Impact on Activities score.

## Data Availability

The data presented in this study are available on reasonable request from the corresponding author.

## Appendix A

Work Satisfaction Scale—Doctor

Based on Zalewska’s work [22]

Below, there are statements that you may agree or disagree with. Using the 1–7 scale below, indicate your agreement with each item by placing the appropriate number on the line preceding that item. Please be honest in your responding, according to the scale:

1. Strongly disagree

2. Disagree

3. Rather disagree

4. Hard to say if I agree or disagree

5. Rather agree

6. Agree

7. Strongly agree

Regarding only your work with the elderly patients (65+), please respond to following statements:

□ My work is close to the ideal in many ways.

□ The conditions of my work are excellent.

□ I’m satisfied with my job.

□ So far, I’ve succeeded in achieving my work goals.

□ If I was to decide again, I would choose the same job.

## Appendix B

PRACTA—Health Impact on Activities (HIA) scale

Please specify to what extent your health limits your ability to maintain the following (one “X” for each statement):

	**Doesn’t Limit at All**	**Limits a Little**	**Limits Moderately**	**Limits Very Much**
Full body hygiene	1□	2□	3□	4□
Dressing	1□	2□	3□	4□
Preparing and eating meals	1□	2□	3□	4□
Medication taking	1□	2□	3□	4□
Moving around the house	1□	2□	3□	4□
Reading and watching TV	1□	2□	3□	4□
Shopping	1□	2□	3□	4□
Commuting	1□	2□	3□	4□
Driving	1□	2□	3□	4□
Dealing with official matters	1□	2□	3□	4□

## Appendix C

The PRACTA Patient’s Satisfaction with a Visit Scale (SVSP)

Would you recommend this doctor to your family/friends?

1□ ------- 2□ ------- 3□ ------- 4□ ------- 5□ ------- 6□ ------- 7□

definitely no                                                                     definitely yes

Would you like to come to this doctor again?

1□ ------- 2□ ------- 3□ ------- 4□ ------- 5□ ------- 6□ ------- 7□

definitely no                                                                     definitely yes

If it was easy to change a clinic—would you still come to this one?

1□ ------- 2□ ------- 3□ ------- 4□ ------- 5□ ------- 6□ ------- 7□

definitel no                                                                     definitely yes

How satisfied are you with this visit at the doctor?

1□ ------- 2□ ------- 3□ ------- 4□ ------- 5□ ------- 6□ ------- 7□

definitel no                                                                     definitely yes

Have your hopes for this visit been fulfilled?

1□ ------- 2□ ------- 3□ ------- 4□ ------- 5□ ------- 6□ ------- 7□

definitely no                                                                     definitely yes

How satisfied are you with the time the doctor has spent on the consultation?

1□ ------- 2□ ------- 3□ ------- 4□ ------- 5□ ------- 6□ ------- 7□

definitely no                                                                     definitely yes

Considering registration, travel, waiting time, help you received, etc., was the visit worth coming for?

1□ ------- 2□ ------- 3□ ------- 4□ ------- 5□ ------- 6□ ------- 7□

definitely no                                                                     definitely yes

## Appendix D

Contextual analysis results

The following are the statistics for the effects of independent variables on CT in three contextual analysis (provided separately for facility-, doctor, and patient-related factors) presented in paragraph 3.2 of the article:

Facility (Wald’s *χ*^2^ = 78.727, *p* < 0.001; the intercept in the model was 177.091, *p* < 0.001): the number of GPs (*χ*^2^ = 8.209, *p* = 0.004, β = −0.117), staff turnover (*χ*^2^ = 19.708, *p* < 0.001, β = −0.973), and time scheduled per visit (χ² = 8.700, *p* = 0.003, β = 0.168).

Doctor: the self-rated health (*χ*^2^ = 26.815, *p* < 0.001, β = 1.018), training in geriatrics (*χ*^2^ = 3.955, *p* = 0.047, β = −0.354), professional seniority (*χ*^2^ = 3.940, 0.047, β = −0.066), seniority within the facility (*χ*^2^ = 11.100, *p* < 0.001, β = −0.035), working hours in the facility (*χ*^2^ = 8.389, *p* = 0.004, β = 0.036), working hours in total (*χ*^2^ = 9.010, *p* = 0.003, β = −0.039), and satisfaction with their work with seniors (*χ*^2^ = 8.977, *p* = 0.003, β = −0.417). That model yielded Wald’s *χ*^2^ = 69.691, *p* < 0.001, with an intercept equal to 130.078, *p* < 0.001.

Patient (Wald’s *χ*^2^ = 75.730, *p* < 0.001 for the model; the intercept in the model was 59.189, *p* < 0.001): their education (*χ*^2^ = 16.119, *p* < 0.001, β = −0.467), hospital use in the last 12 m (*χ*^2^ = 5.820, *p* = 0.016, β = −0.744), HIA (*χ*^2^ = 12.355, *p* < 0.001, β = 0.963), waiting time (*χ*^2^ = 9.172, *p* = 0,002, β = −0.178), and attendance in years (*χ*^2^ = 6.620, *p* = 0.010, β = −0.053).

The following are the statistics for the effects of independent variables on PS in three contextual analyses (provided separately for facility-, doctor, and patient-related factors) presented in paragraph 3.3. of the article:

Facility (Wald’s *χ*^2^ = 177.057, *p* < 0.001; for the model, the intercept = 2274.429, *p* < 0.001): the location (*χ*^2^ = 34.867, *p* < 0.001, β = −0.084), number of GPs employed (*χ*^2^ = 6.717, *p* = 0.01, β = −0.020), and time scheduled for visit (*χ*^2^ = 62.641, *p* < 0.001, β = −0.063).

Doctor (Wald’s *χ*^2^ = 61.942, *p* < 0.001; for the model, the intercept = 703.732, *p* < 0.001): their marital status (*χ*^2^ = 8.651, *p* = 0.034), self-rated health (*χ*^2^ = 7.797, *p* = 0.005, β = −0.091), estimated rate of older patients aged 65+ (*χ*^2^ = 8.729, *p* < 0.001, β = −0.083), seniority in the facility (*χ*^2^ = 4.476, *p* = 0.034, β = 0.005), and satisfaction with work with seniors (*χ*^2^ = 7.681, *p* = 0.006, β = 0.064).

Patient (Wald’s *χ*^2^ = 350.831, *p* < 0.001, for the model, the intercept = 393.723, *p* < 0.001): their education (*χ*^2^ = 7.265, *p* = 0.007, β = −0.043), aim of the visit (*χ*^2^ = 19.208, *p* < 0.001, β = 0.223), hospital use (*χ*^2^ = 5.868, *p* = 0.015, β = −0.110), HIA (*χ*^2^ = 15.341, *p* < 0.001, β = −0.168), waiting time for their visit (*χ*^2^ = 196.314, *p* < 0.001, β = −0.270), easiness of scheduling (*χ*^2^ = 11.215, *p* = 0.001, β = 0.078), frequency of attendance in the last 12 m (*χ*^2^ = 50.044, *p* < 0.001, β = 0.235), and CT (*χ*^2^ = 6.544, *p* = 0.011, β = −0.008).
